# Pulmonary Inflammation Impacts on CYP1A1-Mediated Respiratory Tract DNA Damage Induced by the Carcinogenic Air Pollutant Benzo[*a*]pyrene

**DOI:** 10.1093/toxsci/kfv086

**Published:** 2015-04-23

**Authors:** Volker M. Arlt, Annette M. Krais, Roger W. Godschalk, Yanira Riffo-Vasquez, Iveta Mrizova, Candice A. Roufosse, Charmaine Corbin, Quan Shi, Eva Frei, Marie Stiborova, Frederik-Jan van Schooten, David H. Phillips, Domenico Spina

**Affiliations:** *Analytical and Environmental Sciences Division, MRC-PHE Centre for Environment & Health, King’s College London, London SE1 9NH, United Kingdom,; ^†^Department of Toxicology, School for Nutrition, Toxicology and Metabolism (NUTRIM), Maastricht University Medical Centre, 6200 MD Maastricht, The Netherlands,; ^‡^Sackler Institute of Pulmonary Pharmacology, Institute of Pharmaceutical Science, King's College London, London SE1 9NH, United Kingdom,; ^§^Department of Biochemistry, Faculty of Science, Charles University, 12840 Prague 2, Czech Republic;; ^¶^Department of Histopathology, Imperial College Healthcare NHS Trust, Hammersmith Hospital, London W12 0HS, United Kingdom, and; ^||^Division of Preventive Oncology, National Center for Tumor Diseases, German Cancer Research Center (DKFZ), Im Neuenheimer Feld 280, 69120 Heidelberg, Germany

**Keywords:** benzo[*a*]pyrene, pulmonary inflammation, cytochrome P450, carcinogen metabolism, DNA adducts, bronchoalveolar lavage

## Abstract

Pulmonary inflammation can contribute to the development of lung cancer in humans. We investigated whether pulmonary inflammation alters the genotoxicity of polycyclic aromatic hydrocarbons (PAHs) in the lungs of mice and what mechanisms are involved. To model nonallergic acute inflammation, mice were exposed intranasally to lipopolysaccharide (LPS; 20 µg/mouse) and then instilled intratracheally with benzo[*a*]pyrene (BaP; 0.5 mg/mouse). BaP-DNA adduct levels, measured by ^32^P-postlabeling analysis, were approximately 3-fold higher in the lungs of LPS/BaP-treated mice than in mice treated with BaP alone. Pulmonary Cyp1a1 enzyme activity was decreased in LPS/BaP-treated mice relative to BaP-treated mice suggesting that pulmonary inflammation impacted on BaP-induced Cyp1a1 activity in the lung. Our results showed that Cyp1a1 appears to be important for BaP detoxification *in vivo* and that the decrease of pulmonary Cyp1a1 activity in LPS/BaP-treated mice results in a decrease of pulmonary BaP detoxification, thereby enhancing BaP genotoxicity (ie, DNA adduct formation) in the lung. Because less BaP was detoxified by Cyp1a1 in the lungs of LPS/BaP-treated mice, more BaP circulated via the blood to extrapulmonary tissues relative to mice treated with BaP only. Indeed, we observed higher BaP-DNA adduct levels in livers of LPS/BaP-treated mice compared with BaP-treated mice. Our results indicate that pulmonary inflammation could be a critical determinant in the induction of genotoxicity in the lung by PAHs like BaP. Cyp1a1 appears to be involved in both BaP bioactivation and detoxification although the contribution of other enzymes to BaP-DNA adduct formation in lung and liver under inflammatory conditions remains to be explored.

Globally, lung cancer is the leading cause of cancer death. Tobacco smoking is the overwhelming cause of lung cancer, although vehicle engine exhaust (eg, diesel exhaust) and ambient air pollution are also implicated (IARC, 2013; Loomis *et al.*, 2013). Inflammatory diseases of the lung, including fibrosis and chronic obstructive pulmonary disease (COPD), are associated with higher lung cancer risk (Brody and Spira, 2006; Schottenfeld and Beebe-Dimmer, 2006). Lung cancer risk in smokers with COPD is increased up to 10-fold in comparison to smokers without COPD (Brody and Spira, 2006). Many inflammatory agents can contribute to the development of diseases like COPD or asthma, including inhaled combustion-derived particles such as cigarette smoke, ambient air particulate matter, and diesel exhaust particles (Kelly and Fussell, 2011). Inhalation of such particles can cause a local pulmonary response which is characterized by the influx of neutrophils into the airways (Knaapen *et al.*, 2006). In contrast to their innate protective role in immunity, neutrophils contribute to the pathogenesis of inflammatory lung diseases like COPD and promote tumor development (Grivennikov *et al.*, 2010; Knaapen *et al.*, 2006).

A number of studies have found that occupational exposure to diesel exhaust leads to increased risk of lung cancer (Attfield *et al.*, 2012; Silverman *et al.*, 2012) and the International Agency for Research on Cancer (IARC) has classified diesel engine exhaust as carcinogenic to humans (Group 1) (IARC, 2013). However, the mechanism of diesel carcinogenesis and precise identity of the carcinogenic components of diesel exhaust are still incompletely understood, as is the magnitude of the carcinogenic risk from environmental exposure. Although exposure to diesel exhaust material induces pulmonary inflammation and exacerbates chronic respiratory inflammatory conditions (Kelly and Fussell, 2011), the contribution of such inflammation to diesel exhaust associated carcinogenic risk potential has not been examined in any great detail. By analogy with the causation of lung cancer by tobacco smoking (Walser *et al.*, 2008), it was therefore the aim of this study to examine how inflammation in the lung alters the genotoxicity of polycyclic aromatic hydrocarbons (PAHs), which occur in the particulate phase of diesel exhaust, and what specific mechanisms are involved.

PAHs such as benzo[*a*]pyrene (BaP), also an IARC Group 1 carcinogen (IARC, 2010), exert their carcinogenic effects only after metabolic activation. As shown in Figure 1 BaP is activated by cytochrome P450 (CYP) enzymes, CYP1A1 and CYP1B1 being the most important isoenzymes (Baird *et al.*, 2005), resulting in highly reactive diol-epoxides capable of forming covalent DNA adducts that can lead to mutations through errors in DNA replication (Phillips, 2005). Inflammatory reactions *in vivo* involve the production and release of a range of signaling molecules including cytokines and chemokines (Grivennikov *et al.*, 2010; Schwarze *et al.*, 2013). *In vitro* experiments have shown that cytokines like tumor necrosis factor-α (TNF-α) formed after environmental exposures can alter the expression of metabolic enzymes such as CYPs (eg, CYP1A1, CYP1B1) involved in BaP bioactivation (Smerdova *et al.*, 2014; Umannova *et al.*, 2008). Other *in vitro* studies have revealed that neutrophil-derived myeloperoxidase can activate the BaP metabolite BaP-7,8-dihydrodiol to reactive species (ie, BaP-7,8-dihydrodiol-9,10-epoxide [BPDE]) that form DNA adducts in lung cells (Borm *et al*., 1997; Petruska *et al*., 1992).

**FIG. 1.**
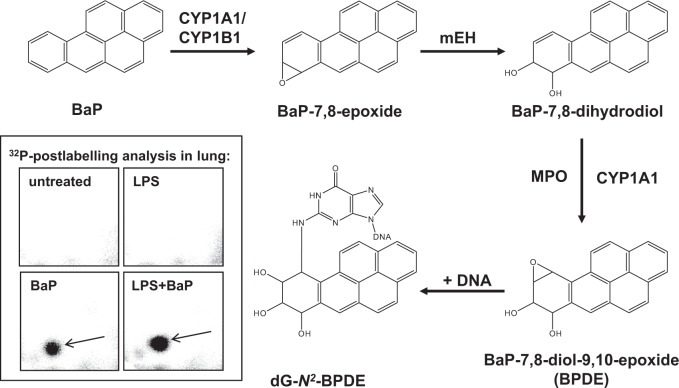
Main metabolic pathway in the bioactivation and DNA adduct formation of BaP in lung. See text for details. CYP, cytochrome P450; mEH, microsomal epoxide hydrolase; MPO, myleoperoxidase. Inserts: autoradiographic profiles of DNA adducts in lungs formed in mice; the origin, at the bottom left-hand corner, was cut off before exposure. Autoradiographic profiles in the lungs are representative of those observed in the livers. The arrow shows 10-(deoxyguanosin-*N*^2^-yl)-7,8,9-trihydroxy-7,8,9,10-tetrahydro-BaP (dG-*N*^2^-BPDE).

In this study, we investigated whether lung inflammation alters the capacity for diesel exhaust carcinogens like BaP to cause DNA damage (eg, DNA adducts) *in vivo* and the mechanisms involved. To model nonallergic acute inflammation, mice were exposed to lipopolysaccharide (LPS) and then instilled with BaP. DNA adduct formation was determined by ^32^P-postlabeling analysis.

## MATERIALS AND METHODS

### 

#### 

##### Carcinogen

BaP (purity ≥96%) was obtained from Sigma Aldrich.

##### Animal treatment

**FIG. 2.**
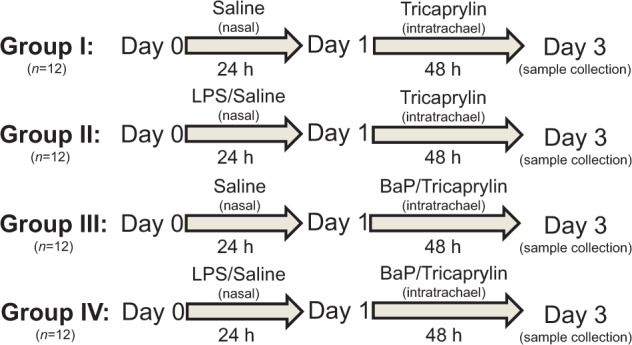
Study design and animal treatment. See Materials and Methods for additional information.

C57B16 mice (male; approximately 8–10 weeks old, 20-25 g) were obtained from Charles River Laboratories. All animal experiments were carried out at King’s College London under license according to protocols approved by the Home Office under “The Animals (Scientific Procedures) Act (1986)” after approval by the institutional ethics committee. Animals were kept under controlled pathogen-free conditions and allowed food and water *ad libitum*. In total, 4 groups of mice (*n* = 4 per experiment; repeated in triplicate; *n* = 12 in total) were used as follows (see Fig. 2): Group I: mice were instilled nasally with saline and 24 h later instilled intratracheally with vehicle, tricaprylin (25 µl/mouse). Group II: to induce acute pulmonary inflammation mice received an intranasal dose of 20 µg LPS (*Escherichia coli*, serotype O55:B5; 1 mg/ml; Sigma), and 24 h later they received tricaprylin (25 µl/mouse) by intratracheal instillation. Group III: mice were instilled nasally with saline and 24 h later instilled intratracheally with BaP (0.5 mg BaP dissolved in 25 µl tricaprylin). Group IV: mice received an intranasal administration of 20 µg LPS followed 24 h later with BaP (0.5 mg BaP/mouse) by intratracheal instillation. In order to have sufficient material available for histopathology and several biological assays, experiments were performed in triplicate on separate occasions (3 × *n* =4/group). All instillations were performed under anesthesia with isoflurane (Sigma) following injection with ketamine/zylazine (1 mg/0.166 mg per mouse, respectively; Sigma). Mice were killed 3 days after exposure after anesthesia with 2 g/kg body weight urethane (Sigma) by intraperitoneal administration and a cannula was inserted into the exposed trachea. For the collection of inflammatory cells by bronchoalveolar lavage (BAL) 3 0.5-ml aliquots of sterile saline were injected into the lungs. Lung and liver tissue were also collected, snap-frozen in liquid nitrogen and stored at −80°C until analysis. For histopathology lung sections were fixed for 48 h in PBS containing 4% paraformaldehyde.

##### Assessment of the pulmonary inflammation by histopathology and BAL analysis

Fixed lung sections were embedded in paraffin and 7-micron sections were cut and stained with hematoxylin–eosin (H&E) (Arlt *et al.*, 2011). Slides were randomized and analyzed at ×10 magnification for the number of fields with inflammation, expressed as percentage of the total number of fields of lung tissue on the section. At ×40 magnification, inflammation was qualified as either predominantly neutrophilic or predominantly monocytic.

From the collected BAL fluid, a 50-µl aliquot was added to 50 µl of hemolysis (Turk) solution. The total number of cells in the BAL fluid was counted with an improved Neubauer hemocytometer. For differential cell counts, cytospin preparations were prepared from aliquots of BAL fluid (100 µl), centrifuged at 250 × g for 1 min using a Shandon Cytospin 2 (Shandon Southern Instruments, Sewickley, Pennsylvania) at room temperature and stained with Diffquick. Two hundred cells were counted to determine the proportion of neutrophils, eosinophils, and monocytes using standard morphological criteria (Holand *et al.*, 2014).

##### Detection of DNA adducts

DNA from tissue was isolated by a standard phenol–chloroform extraction method. DNA adduct analysis was performed by the nuclease P1 enrichment version of the ^32^P-postlabeling method as described previously (Phillips and Arlt, 2007, 2014) with minor modifications. DNA samples (4 μg) were digested with micrococcal nuclease (288 mU; Sigma) and calf spleen phosphodiesterase (1.2 mU; MP Biomedical), and then enriched and labeled as reported. Resolution of ^32^P-labeled adducts was performed by polyethyleneimine-cellulose thin-layer chromatography (TLC) (Arlt *et al.*, 2008). After chromatography TLC plates were scanned using a Packard Instant Imager (Dowers Grove, Illinois). DNA adduct levels (RAL, relative adduct labeling) were calculated from adduct counts per minute, the specific activity of [γ-^32^P]ATP and the amount of DNA (pmol) used. Results were expressed as DNA adducts/10^8^ normal nucleotides (nt). An external BPDE-modified DNA standard was used to identify BaP-DNA adducts.

##### Preparation of pulmonary and hepatic microsomal and cytosolic samples

Pooled pulmonary and hepatic microsomal and cytosolic fractions (*n* = 4) were isolated as described (Arlt *et al.*, 2008; Martin *et al.*, 2010). Briefly, tissue samples were pulverized by grinding snap-frozen pooled lung or liver specimens in a Teflon container frozen in liquid nitrogen with a steel ball using a dismembrator (2600 UPM for 30 s; Braun Melsungen AG, Germany). The frozen tissue powder was then homogenized by hand in.067 M potassium phosphate buffer (pH 7.4) with 0.5% potassium chloride in a Potter-Elvehjem glass-Teflon homogenizer. The buffer volume (in µl) used was 3 times the weight (in mg) of the organ. Nuclei and debris were removed by centrifugation at 18 × g for 30 min at 4°C. From the supernatant, microsomal pellets were obtained at 100 000 *g* after 1 h. Supernatant (cytosolic fraction) was gently levered off the sediment into 200-µl aliquots and stored at −80°C until further analysis. The sediment (microsomal fraction) was resuspended in phosphate buffer (lung in approximately the same volume (in µl) as their weight (in mg), liver in twice their weight) and small aliquots (100 µl) were stored at −80°C until further analysis. Protein concentrations in cytosolic and microsomal fractions were measured using the bicinchoninic acid protein assay with bovine serum albumin as a standard.

##### Expression of pulmonary and hepatic Cyp1 protein

Immunoquantitation of Cyp1a1 and Cyp1b1 in microsomal fractions was carried out by sodium dodecyl sulfate-10% polyacrylamide gel electrophoresis of samples containing 30 μg microsomal proteins. After migration, proteins were transferred onto polyvinylidenedifluoride membranes. Mouse Cyp1a1 protein was probed with goat-anti rat CYP1A1 polyclonal antibodies (1:2500, Antibodies-online GmbH, Aachen, Germany) as reported elsewhere (Stiborova *et al.*, 2014). The goat-anti rat CYP1A1 antibodies recognize this enzyme in mouse pulmonary and hepatic microsomes as 1 protein band. Rat recombinant CYP1A1 (in Supersomes, Gentest Corp., Woburn, Massachusetts) was used as positive controls to identify the band of Cyp1a1 in murine microsomes. Mouse Cyp1b1 protein was probed with rabbit-anti human CYP1B1 polyclonal antibodies (G-25) (1:200, Santa Cruz Biotechnology, Dallas, Texas). The goat-anti rabbit CYP1B1 antibodies recognize this enzyme as 1 protein band. Human recombinant CYP1B1 (in Supersomes) was used as positive control. The antigen–antibody complex was visualized with an alkaline phosphatase-conjugated rabbit anti-chicken IgG antibody and 5-bromo-4-chloro-3-indolylphosphate/nitrobluetetrazolium as chromogenic substrate (Stiborova *et al.*, 2006). Glyceraldehyde phosphate dehydrogenase was used as loading control and detected by its antibody (1:750, Millipore, Massachusetts). Band intensity was quantified using the GeneTools software.

##### Measurement of pulmonary and hepatic Cyp1a enzyme activity

Microsomal Cyp1a enzyme activity (measured as relative fluorescence unit [RFU]/minute) was determined by following the conversion of 7-ethoxyresorufin into resorufin (EROD assay) using fluorescent measurement on a Synergy HT Plate Reader (Bio-TEK Instruments; 530 nm excitation, 580 nm emission) (Mizerovska *et al.*, 2011). Cyp1a enzyme activity (measured as RFU/minute) was also measured with 3-cyano-7-ethoxycoumarin (CEC) as substrate (Martin *et al.*, 2010). Briefly, in a 96-well plate the incubation mixture (200 µl) contained 67 mM potassium phosphate buffer (pH 7.4), 9 mM glucose-6-phosphate, 0.9 U glucose-6-phosphate dehydrogenase, 4.5 mM magnesium chloride, 0.9 mM NADP, 5 µM CEC (dissolved in DMSO (dimethyl-sulfoxide)), and 50 µg of microsomal fraction. The reaction was initiated by the addition of CEC and the formation of 3-cyano-7-hydroxycoumarin was measured every 2 min for 30 min (409 nm excitation, 460 nm emission).

##### Measurement of pulmonary and hepatic Nqo1 enzyme activity

Nqo1 enzyme activity in cytosolic samples was measured with menadione (2-methyl-1,4-naphthoquinone) as substrate essentially as described previously (Mizerovska *et al.*, 2011). The standard assay system in a 24-well plate contained in 1 ml (final concentration) 25 mM Tris-HCl (pH 7.5), 0.12 % bovine serum albumin, 200 μM NADH, 10 μM menadione (dissolved in methanol), 77 μM cytochrome *c*, and 50 µg of cytosolic fraction. The reaction was initiated by the addition of the cytosolic fraction. Enzyme activity (measured as RFU/min) was determined by following the conversion of cytochrome *c* at 550 nm on a Synergy HT Plate Reader.

##### Expression of Cyp1b1 gene expression in the lung

Gene expression analysis was essentially performed as described (Krais *et al.*, 2015). Briefly, RNA was isolated from lung samples using the GenElute Mammalian Total RNA Mini Prep Kit (Sigma, UK) according to the manufacturer’s instruction. Reserve transcription was performed using random primers and SuperScript III Reserve Transcriptase (Life Technologies, UK) RNA expression was analyzed by quantitative real-time polymerase chain reaction (qRT-PCR) using TaqMan Universal PCR Master Mix (Life Technologies) and TaqMan gene expression primers according to the manufacturer’s protocol with a 7500HT Fast Real Time PCR System (Applied Biosystems, UK). Probe (Life Technologies) *Cyp1b1*-Mm00487229_m1 was used and expression levels were normalized to housekeeping gene *Gapdh* (4352341E). Relative gene expression was calculated using the comparative threshold cycle (*C_T_*) method (Kucab *et al.*, 2012).

##### Measurement of nucleotide excision repair capacity

The ability of nucleotide excision repair (NER)-related enzymes present in isolated tissue extracts to detect and incise substrate DNA containing BPDE-DNA adducts was measured using a modified comet assay (Langie *et al.*, 2006). Tissue protein extracts were prepared as described previously (Gungor *et al.*, 2010a), and protein concentrations were optimized for analysis of lung and liver samples (0.2 mg/ml). The *ex vivo* repair incubation and electrophoresis were performed according to the published protocol (Langie *et al.*, 2006). Dried slides stained with ethidium bromide (10 µg/ml) were viewed with a Zeiss Axioskop fluorescence microscope. Comets were scored using the Comet III system (Perceptive Instruments, UK). Fifty nucleoids were assessed per slide and each sample was analyzed in duplicate. All samples were measured blindly. Tail intensity (% tail DNA) was used to calculate repair capacity of the tissue extracts (Langie *et al.*, 2006).

##### Statistical analysis

Statistical analyses were performed with Prism GraphPad Software and *P* < .05 was considered significant.

## RESULTS

### 

#### 

##### Pulmonary histopathology

**FIG. 3.**
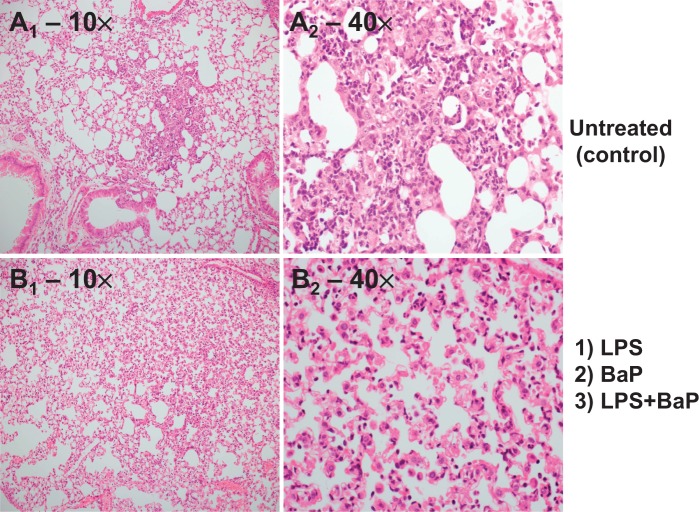
Histological analysis of pulmonary inflammation. Representative photomicrographs of lung tissue section stained with H&E. A, Control mice: small sense foci of predominantly neutrophils; B, LPS-, BaP-, or LPS + BaP-treated lung: large loose foci of predominantly monocytes. Original magnification ×10, left panel; ×40, right panel. Semiquantitative assessment of pulmonary inflammation is summarized in Table 1.

Pulmonary inflammation 3 days after exposure to LPS was assessed by H&E staining (Fig. 3). The semiquantitative assessment is summarized in Table 1. The bronchi and vessels in all groups appeared unaffected. In all 4 groups, there were foci of alveolar inflammation (pneumonia), but the size of the foci and the composition of inflammatory cells were different. Controls (Group I) showed few inflammatory foci (5%–14% of fields), which were small in size and composed predominantly of neutrophils. LPS-treated animals (Group II) showed an increase in inflammatory foci (26%–92% of fields) as loose collections mainly of macrophages extending over a larger area. BaP and LPS + BaP treated animals (Groups III and IV) showed an intermediate number of inflammatory foci (4%–32% and 4%–41% respectively), roughly of the same composition and size as seen in the LPS-treated animals (Group II).

**TABLE 1.** Semiquantitative Assessment of Pulmonary Inflammation From H&E Staining of Lung Sections

Treatment Group (*n* = 4 per group)	Percentage of Fields with Inflammation (median)	Size of Inflammatory Foci	Predominant Cell Type
Controls (Group I)	5-14 (6.5)	Small dense	Neutrophils
LPS (Group II)	26-92 (79)	Large loose	Monocytes
BaP (Group III)	4-32 (12.5)	Large loose	Monocytes
LPS + BaP (Group IV)	4-41 (18.5)	Large loose	Monocytes

##### Inflammatory response in BAL

**FIG. 4.**
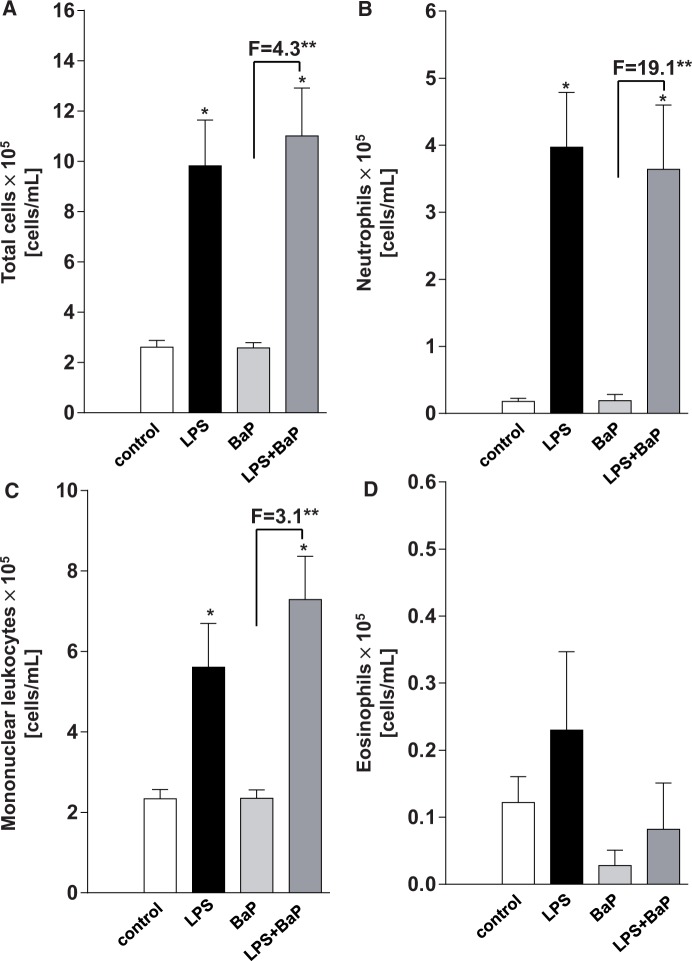
Effect of BaP treatment on pulmonary inflammation assessing bronchoalveolar lavage fluid. Total (A), neutrophil (B), mononuclear leukocytes (C), and eosinophil (D) cells were quantified by hemocytometry from mice treated with LPS, BaP, LPS + BaP, or vehicle only (control). All values are given as the means ± SEM (*n* = 12 per group). In the figure F = fold difference in cell number in LPS/BaP group compared with cell number in BaP group. Statistical analysis was performed by 2-way ANOVA followed by Tukey’s multiple comparisons test (*P < .05, vs control [untreated] mice; **P < .05, different BaP only treated mice).

Using morphological criteria the number of monocytes, eosinophils, and neutrophils were counted in BAL fluid (Fig. 4). LPS treatment (Group II) caused significant increases in neutrophils (Fig. 4B) and mononuclear leucocytes (Fig. 4C) recruitment to the lung relative to control mice (Group I). No such effect was seen for eosinophils (Fig. 4D). The recruitment of neutrophils, used as a measure of pulmonary inflammation, in mice treated with LPS and LPS/BaPwas high (Fig. 4B). In LPS-treated mice (Group II) the number of neutrophils was approximately 22-fold higher than in control mice (Group I) and BaP-treated mice (Group III), however, additional treatment with BaP (Group IV) had no additional effect on neutrophil recruitment. More specifically, 2-way ANOVA showed a statistically significant effect of LPS-induced inflammation on neutrophil recruitment [F(1,41) = 31.11, *P* < .0001] but was not affected by BaP treatment. There was no significant interaction effect.

##### DNA adduct formation in lung and liver

**FIG. 5.**
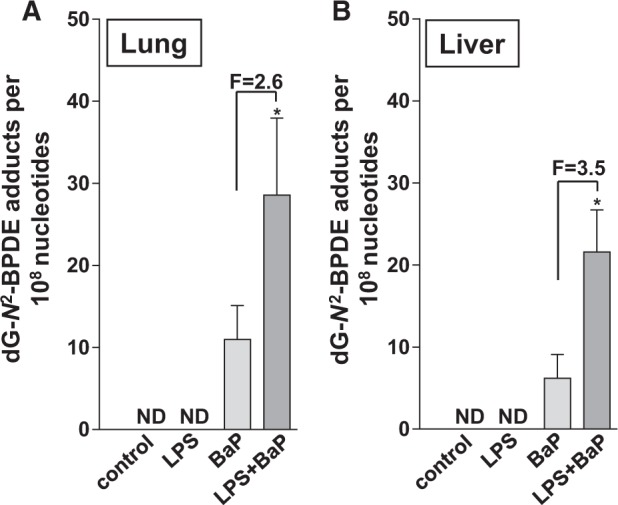
BaP-DNA adduct formation. DNA adduct levels (RAL, relative adduct labeling) were measured by ^32^P-postlabeling in lung (A) and liver (B) of mice treated with LPS, BaP, LPS + BaP, or vehicle only (control). All values are given as the means ± SD (*n* = 4 per group). ND, not detected. In the figure F = fold difference in DNA binding in LPS/BaP group compared with DNA binding in BaP group. Statistical analysis was performed by unpaired 2-tailed *t* test (*P < .05, vs BaP only treated mice).

The DNA adduct pattern observed by TLC ^32^P-postlabeling in BaP-treated mice (Groups III and IV) consisted of a single adduct spot, in both lung and liver. Although the ^32^P-postlabeling method does not provide any structural information of the BaP-derived DNA adduct formed, using mass spectrometry the adduct formed *in vivo* was previously identified (Arlt *et al.*, 2008) as 10-(deoxyguanosin-*N*^2^-yl)-7,8,9-trihydroxy-7,8,9,10-tetrahydro-BaP (dG-*N*^2^-BPDE) (Fig. 1; inserts). DNA adducts were not detected either in control (Group I) or in LPS-treated animals (Group II). BaP-DNA adduct levels ranged from 10 to 30 adducts per 10^8 ^nt (Fig. 5). Adduct levels were significantly higher in both lung (approximately 2.5-fold) and liver (approximately 3.5-fold) of LPS/BaP-treated mice (Group IV) than in mice treated with BaP alone (Group III).

##### Expression of BaP-metabolizing enzymes in lung and liver

**FIG. 6.**
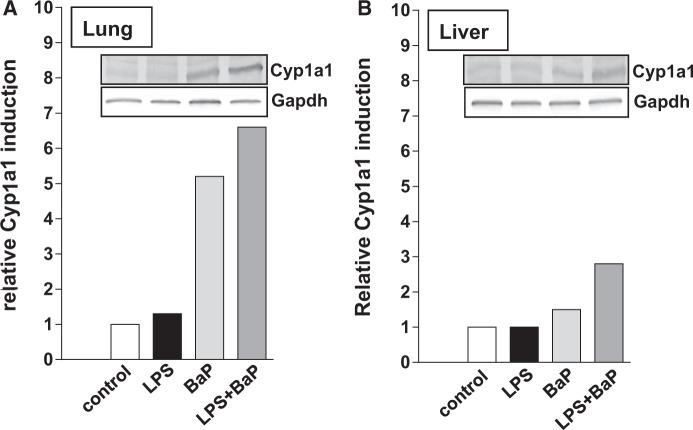
Western blot analysis of Cyp1a1 protein expression in lung (A) and liver (B) of mice treated with LPS, BaP, LPS + BaP, or vehicle only (control). Representative images of the Western blotting are shown; duplicate analysis was performed on separate occasions. Gapdh protein expression was used as loading control.

**FIG. 7.**
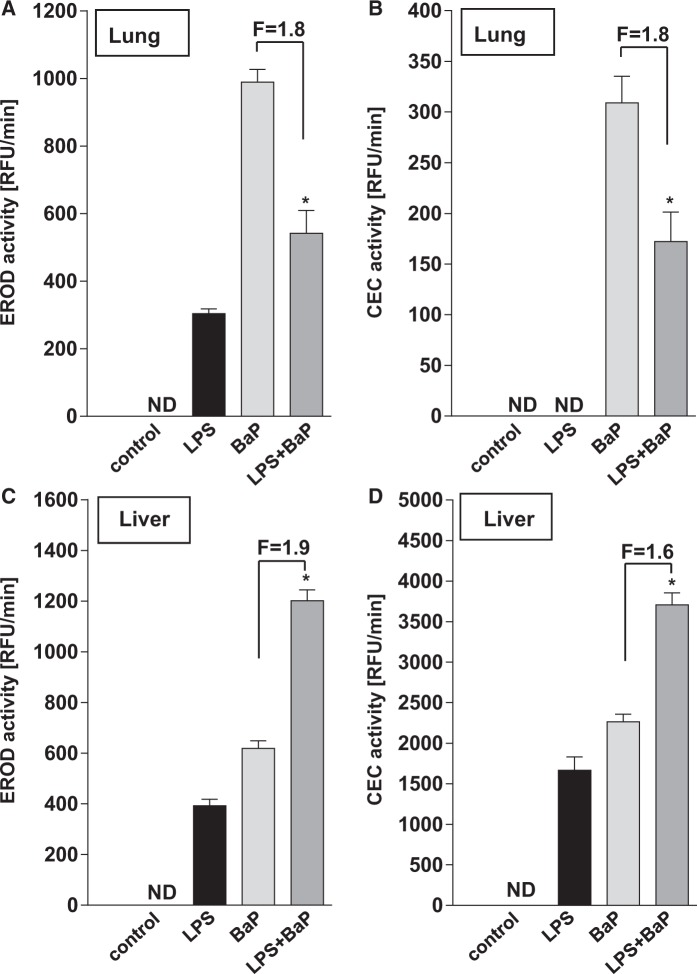
Effect of BaP treatment on Cyp1a enzyme activity. Cyp1a1 enzyme activity as measured by EROD (A + C) or CEC activity (B + D) in microsomal fractions isolated from lung (A + B) or liver (C + D) tissues of mice treated with LPS, BaP, LPS + BaP, or vehicle only (control). All values are given as the means ± SD of 3 separate determinations. RFU, relative fluorescence unit. ND, not detected. In the figure F = fold difference in enzyme activity in LPS/BaP group compared with enzyme activity in BaP group. Statistical analysis was performed by unpaired 2-tailed *t* test (*P < .05, vs BaP only treated mice).

Cyp1a1 protein levels measured by Western blotting showed a approximately 5-fold induction in the lungs after BaP treatment (Group III) (Fig. 6A). Similarly pulmonary Cyp1a1 protein levels increased approximately 5-fold in LPS/BaP-treated mice (Group IV) relative to mice treated with LPS alone (Group II). Even though intensities of the Cyp1a1 protein bands in control (untreated) and LPS-treated mice were weak, a clear increase in Cyp1a1 protein levels was detectable in BaP- and LPS/BaP-treated mice. In accordance with these findings treatment of mice with BaP led to a strong increase in EROD (Fig. 7A) and CEC activity (Fig. 7B) in pulmonary microsomes. Interestingly, pulmonary Cyp1a enzyme activity was significantly lower (approximately 2-fold) in LPS/BaP-treated mice (Group IV) than in mice treated with BaP alone (Group III).

Using Western blotting, we found only a slight increase (approximately 1.5-fold) in Cyp1a1 protein levels in the liver after BaP treatment (Group III) (Fig. 6B). Hepatic Cyp1a1 protein levels increased further in the LPS/BaP-treated mice (Group IV) relative to mice treated with BaP only (Group III). Similarly, hepatic EROD (Fig. 7C) and CEC activity (Fig. 7D) was up to approximately 2-fold higher in LPS/BaP-treated mice (Group IV) compared with mice treated with BaP only (Group III). In addition, LPS exposure alone let to a detectable Cyp1a1 activity in the liver with both substrates (Group II).

**FIG. 8.**
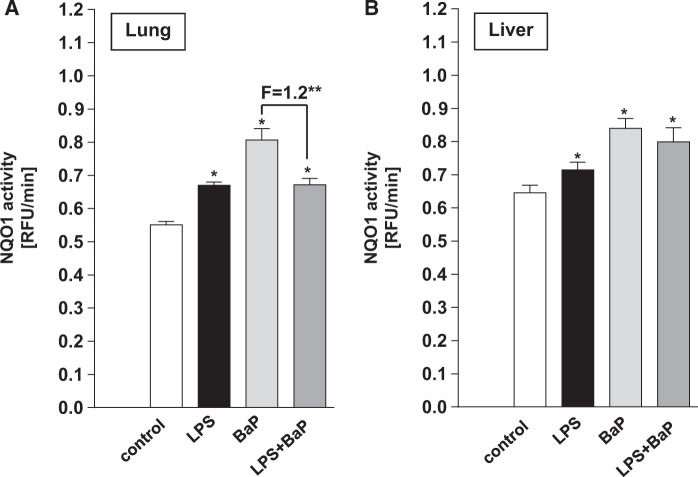
Effect of BaP treatment on Nqo1 enzyme activity. Nqo1 enzyme activity was measured in cytosolic fractions isolated from lung (A) or liver (B) tissues of mice treated with LPS, BaP, LPS + BaP, or vehicle only (control). All values are given as the means ± SD of 3 separate determinations. RFU, relative fluorescence unit. In the figure F = fold difference in enzyme activity in LPS/BaP group compared with enzyme activity in BaP group. Statistical analysis was performed by 2-way ANOVA followed by Tukey’s multiple comparisons test (*P < .05 vs control [untreated] mice; **P < .05, vs BaP only treated mice).

As BaP derivates can also be partly metabolized by NQO1, we also determined the activity of Nqo1 in mice exposed to BaP. Nqo1 activity was detected in both lung and liver cytosolic samples of all groups (Fig. 8). Nqo1 enzyme activity was higher after LPS (Group II), BaP (Group III) and LPS/BaP exposure (Group IV) relative to controls (Group I), in both lung (Fig. 8A) and liver (Fig. 8B). Interestingly, pulmonary Nqo1 enzyme activity was significantly lower in LPS/BaP-treated mice (Group IV) than mice treated with BaP alone (Group III), although the magnitude of the effect was modest (1.2-fold) (Fig. 8A). No difference in Nqo1 enzyme activity between the BaP (Group III) and LPS/BaP group (Group IV) was observed in the liver (Fig. 8B).

**FIG. 9.**
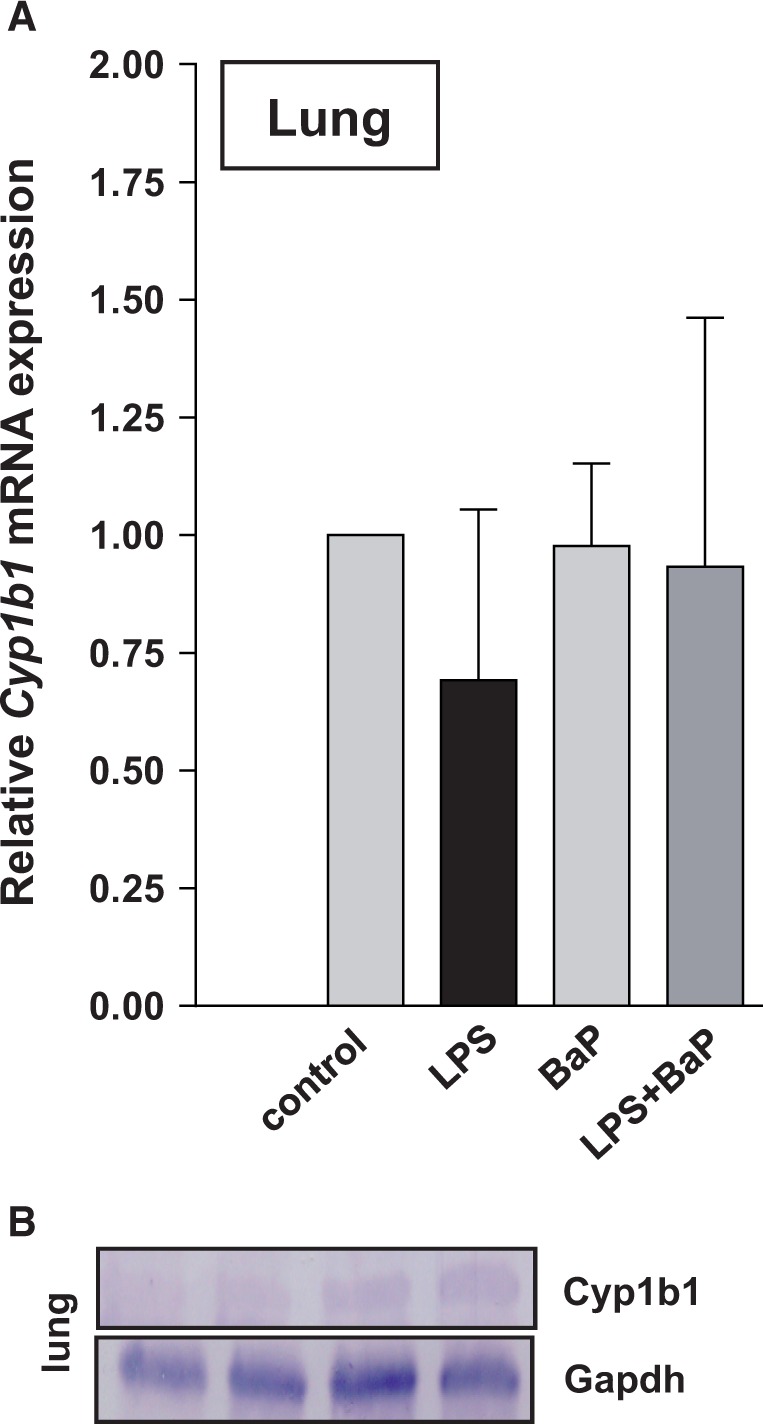
Expression of Cyp1b1 in the lung of mice treated with LPS, BaP, LPS + BaP, or vehicle only (control). A, Gene expression of *Cyp1b1* assessed by RT-PCR. All values are given as the means ± SD (*n* = 4 per group). For statistical analysis, the relative mRNA expression data were log2 transformed and analyzed using a single sample *t* test with Bonferroni correction against the population control mean of 0; no significant differences were observed. B, Cyp1b1 protein expression determined by Western blot analysis. Representative images are shown; duplicate analysis was performed on separate occasions. Gapdh protein expression was used as loading control.

As previous studies have indicated that CYP1B1 may play a role in the metabolic activation of BaP within inflamed tissue (Smerdova *et al.*, 2013, 2014; Umannova *et al.*, 2008), expression of *Cyp1b1* mRNA in the lung was determined by qRT-PCR. However, as shown in Figure 9A no difference in *Cyp1b1* expression was observed between treatment groups. These results were in line with Cyp1b1 protein expression determined in pulmonary microsomes (Fig. 9B). Only very faint Cyp1b1 protein bands were detectable by Western blotting in all treatment groups (Groups I–IV) which could not be accurately quantified.

##### DNA repair capacity in lung and liver

**FIG. 10.**
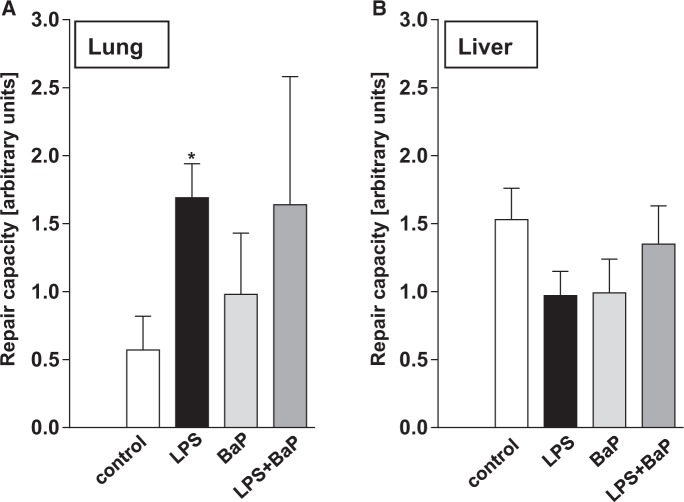
NER repair capacity was measured in tissue extracts isolated from lung (A) or liver (B) tissues of mice treated with LPS, BaP, LPS + BaP, or vehicle only (control). All values are given as the means ± SD (*n* = 3 per group). For statistical analysis, the relative repair capacity data were log-transformed and analyzed by 2-way ANOVA followed by Sidak’s multiple comparisons test (*P < .05, vs control [untreated] mice).

We assessed whether pulmonary inflammation had an influence on NER activity. We found that in the lung the repair capacity was higher (approximately 4-fold) in LPS-treated mice (Group II) than in controls (Group I) (Fig. 10A). More specifically, 2-way ANOVA of the log-transformed data indicated that pulmonary repair capacity was significantly increased following LPS-induced inflammation [F(1,8) = 10.9, *P* = .0131] (Group II) but was not further affected by BaP treatment (Groups III and IV). There was no significant interaction effect.

BaP treatment alone (Group III) had no effect on NER activity. Pulmonary repair capacities in the LPS (Group II) and LPS/BaP (Group IV) groups were similar to each other but not significantly different in the LPS/BaP group (Group IV) relative to controls (Group I) due to large interindividual variability (Fig. 10A). In the liver no significant changes in NER capacity were observed between groups (Fig. 10B).

## DISCUSSION

In this study, we have shown that pulmonary inflammation modulates the bioactivation of BaP and the concomitant respiratory tract DNA damage induced by it. To induce pulmonary inflammation we treated mice with LPS which is an established model to study non-allergic inflammation (Gungor *et al*., 2010a; Medan *et al*., 2002; Moriya *et al*., 2012). The BaP dose used in this study (0.5 mg/mouse) has been shown to induce mutagenicity in the lung of *gpt* delta mice after a single intratracheal instillation (Hashimoto *et al.*, 2005). We found that BaP-induced DNA adduct formation in the lung was approximately 3-fold higher in LPS/BaP-treated mice compared with mice treated with BaP alone. Considering that inhaled combustion-derived particles such as cigarette smoke, ambient air particulate matter, and diesel exhaust particles contribute to pulmonary inflammation in humans (Kelly and Fussell, 2011) our results demonstrate that pulmonary inflammation could be a critical determinant in the induction of genotoxicity in the lung by particle-bound PAHs like BaP.

Pulmonary inflammation induced by LPS initiates the synthesis of proinflammatory cytokines (Gungor *et al.*, 2010b; Holand *et al.*, 2014). It has been shown that LPS-induced expression of cytokines like TNF-α and interleukin-1β in the liver is associated with altered *CYP* gene expression and CYP enzymes activities during inflammation (Warren *et al.*, 1999). In particular, it has been observed that *Cyp1a1* gene expression is suppressed by LPS and TNF-α in mouse liver and that activation of the nuclear factor-κB (NF-κB) plays an important role in *Cyp1a1* suppression (Ke *et al.*, 2001; Zordoky and El-Kadi, 2009). LPS-mediated decrease of hepatic Cyp1a1 was enhanced and accelerated in mice that lack the aryl hydrocarbon receptor (AhR) (ie, *AhR(*−*/−)* mice) compared with *AhR(+/+)* mice (Wu *et al.*, 2011). Others have shown that enhanced expression of AhR in the thymus of LPS-treated mice was accompanied by increased Cyp1a1 expression which could be repressed by inhibition of NF-κB (Vogel *et al.*, 2014). Further, induction of Cyp1a1 by LPS in the thymus depended on functional AhR as shown in *AhR(*−*/*−*)* mice (Vogel *et al.*, 2014). Together these data show that there is a cross-talk between AhR and inflammatory response that can be critical for the expression of CYP1A1 (Vondracek *et al.*, 2011). However, the observed responses are complex and tissue-specific, but it is noteworthy that PAHs like BaP can induce *Cyp1a1* transcription through binding to AhR (Shimizu *et al.*, 2000; Wang *et al.*, 2011).

In this study, we found a clear induction of Cyp1a1 protein in the lungs after BaP treatment both alone and in combination with LPS. In contrast no change of pulmonary Cyp1a1 protein was observed after LPS treatment alone. Interestingly, pulmonary Cyp1a1 enzyme activity was lower in LPS/BaP-treated mice than in mice treated with BaP alone suggesting that pulmonary inflammation impacted on BaP-induced Cyp1a1 enzyme activity in the lung. Because BaP-DNA adduct levels in the lung were increased in LPS/BaP-treated mice compared with BaP-treated mice this observation may appear puzzling at first. However, previous studies (Arlt *et al.*, 2008, 2012; Nebert *et al.*, 2013) have revealed a paradox, whereby CYP enzymes (particularly CYP1A1) appear to be more important for detoxification of BaP *in vivo*, despite being involved in its metabolic activation *in vitro*. Therefore, the decrease in pulmonary Cyp1a1 enzyme activity in LPS/BaP-treated mice relative to BaP-treated mice, as measured in pulmonary microsomes, led to a decrease in BaP detoxification, thereby enhancing BaP genotoxicity (ie, DNA adduct formation) in the lung. It remains to be investigated how pulmonary inflammation really impacts on Cyp1a1 enzyme activity but not Cyp1a1 protein expression (see below). Some other studies have suggested that CYP1B1 could play a role in the bioactivation of BaP within inflamed tissue as CYP1B1 can be up-regulated by proinflammatory cytokines (ie, TNF-α) in BaP-treated cells *in vitro* and thus may redirect BaP metabolism to form higher amounts of BPDE and to potentiate DNA adduct formation (Smerdova *et al.*, 2013, 2014; Umannova *et al.*, 2008). However, *Cyp1b1* gene expression and Cyp1b1 protein analysis in the lung provided no evidence for an impact of pulmonary inflammation on Cyp1b1-mediated BaP bioactivation *in vivo*.

One mediator that may be involved in the suppression of pulmonary Cyp1a1 enzyme activity after LPS challenge could be the formation of reactive oxygen species (ROS; Morel and Barouki, 1999). In this context it is noteworthy that CYP1A1 can produce ROS during its catalytic cycle (Morel and Barouki, 1999). It has been shown not only that LPS results in increased ROS production but also that ROS suppresses CYP1A1 expression in cultured human cells *in vitro* (Morel and Barouki, 1998). Therefore, it has been proposed that ROS such as hydrogen peroxide are involved in hemoprotein inactivation followed by heme loss (Karuzina and Archakov, 1994a,b). Similarly, BaP *o*-quinones formed during BaP metabolism have been shown to generate ROS (Park *et al.*, 2009). Other potential mechanisms might involve the modification of certain amino acids at or near the active centre of the CYP1A1 enzyme by hydrogen peroxide (Karuzina and Archakov, 1994b). Importantly, inactivated Cyp1a1 protein will retain the epitope for its recognition when assayed by Western blot analysis (El-Kadi *et al.*, 2000) but Cyp1a1 enzyme activity will be lost. Therefore, despite the induction of pulmonary Cyp1a1 protein, as measured by Western blotting in the LPS/BaP-treated mice, we propose that ROS formation leads to an inhibition of Cyp1a1 enzyme activity under the present experimental conditions.

If BaP is detoxified more slowly by Cyp1a1 in the lungs of LPS/BaP-treated mice, it would be predicted that more BaP circulates via the blood to extra-pulmonary tissues in these mice relative to mice treated with BaP alone. Indeed, we observed higher BaP-DNA adduct levels in the livers of LPS/BaP-treated mice compared with BaP-treated mice. Further, it would be predicted that if in LPS/BaP-treated mice more BaP is transported from the lung via the blood to the liver than in BaP-treated mice, induction of Cyp1a1 protein and Cyp1a1 enzyme activity should be higher in the livers of LPS/BaP-treated mice relative to mice treated with BaP alone. Indeed, we found Cyp1a1 protein induction as well as an increase in Cyp1a1 enzyme activity in the livers of LPS/BaP-treated mice compared with BaP-treated mice, as measured in hepatic microsomes. Thus it would appear that a higher circulation of BaP to the liver results in higher DNA adduct levels, overriding the tendency of increased Cyp1a1 enzyme activity to result in a greater capacity to detoxify BaP. Our results suggest a dual role of Cyp1a1 in both bioactivation and detoxification of BaP *in vivo*. Similarly, a dual role of CYP1A1 has been shown in the metabolism of the plant carcinogen aristolochic acid I (AAI) where CYP1A1 is able to catalyze the reductive activation of AAI to *N*-hydroxyaristolactam I and the oxidative detoxification to 8-hydroxyaristolochic acid (Stiborova *et al.*, 2012, 2014). These results in the liver also indicate that the presence of acute inflammation in 1 organ (ie, lung) can influence the bioavailability of PAHs like BaP in other organs (ie, liver) suggesting a systemic effect.

LPS-induced pulmonary inflammation also impacted on the expression of other xenobiotic-metabolizing enzymes such as Nqo1 which may be important as BaP derivatives can be metabolized by NQO1 (Joseph and Jaiswal, 1994, 1998; Shen *et al.*, 2010). We found that Nqo1 enzyme activity was increased in the lung, after LPS and BaP treatment both alone and in combination. This may be critical for the bioactivation of diesel exhaust particle-bound nitro-PAHs as NQO1 has been shown to be a key enzyme in the metabolic activation of nitro-PAHs (Purohit and Basu, 2000; Stiborova *et al.*, 2010). Interestingly, pulmonary Nqo1 enzyme activity was decreased in LPS/BaP-treated mice relative to BaP-treated mice suggesting that the bioactivation of nitro-PAHs would be suppressed in these animals.

NER is considered to be the main DNA repair pathway for bulky DNA adducts (Friedberg, 2001). Using a modified comet assay, we showed that tissue-specific NER capacity did not contribute to the higher BaP-DNA adduct levels observed in LPS/BaP-treated mice than in BaP-treated mice in either lung or liver. LPS treatment led to a significant increase (approximately 4-fold) in NER capacity in the lung. In contrast, Gungor *et al.* (2010a) found that LPS exposure reduced NER capacity in lung tissue homogenate by approximately 50%. Although the LPS dose used was the same in both studies the discrepancy between the 2 studies might be attributable to the different LPS administration regimes (intratracheal instillation vs intranasal administration) but otherwise it remains unexplained at present.

In summary we found that pulmonary inflammation can impact on enzymes (eg, CYPs) involved the activation and detoxification of PAHs. Our findings suggest that inflammatory signals and carcinogenic PAHs like BaP may interact and that LPS-induced pulmonary inflammation inhibits Cyp1a1 enzyme activity, which leads to increased DNA damage through the enhanced formation of covalent BaP-DNA adducts in the lungs *in vivo*. Thus pulmonary inflammation could be a critical contributor to the induction of genotoxicity by particle-bound PAHs in the lung.

## FUNDING

Work at King’s College London is supported by Cancer Research UK (Grant C313/A14329) and the Wellcome Trust (Grants 101 126/Z/12/Z and 101 126/B/13/Z). A.M.K. was supported by a fellowship from the German Research Foundation (DFG). Work at Charles University was supported by the Czech Science Foundation (Grant 15-02328S). C.C. was supported by the MSc Program in Biomedical and Molecular Sciences Research at King’s College London. Q.S. is supported by a personal grant from the Chinese Scholarship Council (CSC).
